# Communication Rate Increase in Drill Strings of Oil and Gas Wells Using Multiple Actuators

**DOI:** 10.3390/s19061337

**Published:** 2019-03-17

**Authors:** Erjian Zhang, Ali Abdi

**Affiliations:** Center for Wireless Information Processing, Department of Electrical and Computer Engineering, New Jersey Institute of Technology, 323 King Blvd, Newark, NJ 07102, USA; ez7@njit.edu

**Keywords:** oil and gas wells telemetry, oil and gas wells communication, measurement while drilling, drill strings, pipes, wireless telemetry, strain sensors, accelerometers, acceleration sensors, actuators

## Abstract

Wireless data communication and telemetry during drilling deep oil and gas wells are important enablers for safe and timely drilling operations. The transmission of information through drill strings and pipes using sound waves is a useful and practical approach. However, given the limited available bandwidth, transmission rates are typically smaller than what is needed. In this paper, a new method and system are proposed to increase the transmission rate over the same bandwidth, by deploying more than one actuator. Upon using multiple actuators, several data streams can be transmitted simultaneously. This increases the data rate without the need for additional bandwidth. The experimental results of a testbed with two actuators are presented, where the transmission rate is doubled with no bandwidth increase. A strain sensor receiver and accelerometer receivers are used to separate and demodulate the two data streams. It is demonstrated that it is possible to recover the data in the new faster system benefiting from two actuators, while having about the same bit error probability performance as a one-actuator system. Various combinations of strain and acceleration sensors are considered at the receive side. Due to some properties of strain channels (e.g., smaller delay spreads and their less-frequency-selective behavior) presented in this paper, it appears that a strain sensor receiver and an accelerometer receiver together can offer a good performance when separating and demodulating the two actuators’ data in the testbed. Overall, the experimental results from the proposed system suggest that upon using more than one actuator, it is feasible to increase the data rate over the limited bandwidth of pipes and drill strings.

## 1. Introduction

When drilling to reach underground oil and gas reservoirs, drilling operators on the ground need to have information on parameters such as temperature, pressure, etc. at the bottom of wellbores. This is crucial for the safe and timely drilling of wells. Such parameters are measured using some sensors downhole and are communicated to surface platforms via a variety of techniques [1]. Mud pulses, electromagnetic waves and acoustic waves have been used for information transmission. For a review and comparison of different methods, interested readers can refer to [2]. The data rate of mud pulses is usually only a few bits/s only, whereas electromagnetic waves experience strong attenuation. The acoustic transmission of information through drill strings is a viable and useful method. However, the limited available bandwidth [3] is a factor limiting the achievable data rates.

In this paper, the key idea is to use multiple actuators to transmit several data streams simultaneously over the same bandwidth, and then separate and demodulate them on the receiving side. This allows for data rate increases and optimal utilization of the small available bandwidth. In fact, in this paper it is shown that by using two actuators on a drill string testbed, the data rate can be doubled by transmitting two data streams simultaneously without increasing the bandwidth. While two piezoelectric actuators were used here, one can use magnetostrictive actuators [4,5,6] as well.

The rest of this paper is organized as follows. The experimental testbed is explained in Section 2. Channel measurements and communication test results are presented in Section 3 and Section 4, respectively. Section 5 provides some concluding remarks.

## 2. The Experimental Testbed

**Figure 1:**
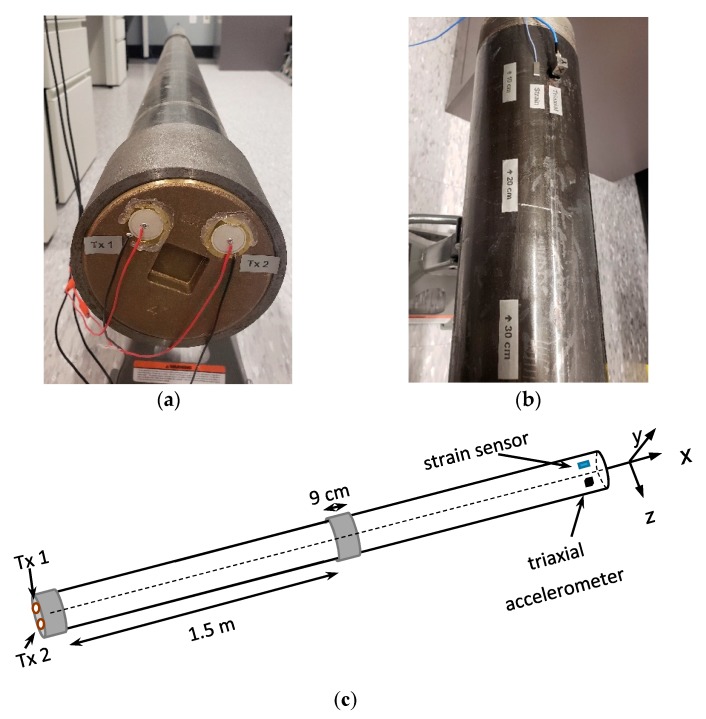
The drill string testbed: (**a**) two piezoelectric transmitters labeled as Tx 1 and Tx 2; (**b**) two receiver sensors, including one strain sensor labeled as Strain, and one triaxial accelerometer labeled as Triaxial; (**c**) schematic drawing of the testbed.

The drill string testbed shown in Figure 1a was composed of two steel pipes connected using a coupling. The length and diameter of each pipe were about 1.5 m and 10 cm, respectively, whereas the length of the coupling was about 9 cm. The two transmitters were piezoelectric transducers, shown in Figure 1a. The receivers are shown in Figure 1b and included one strain sensor (PCB model 740B02), and one triaxial accelerometer (PCB model 356B21). These two sensors are needed to separate and demodulate the two data streams sent out simultaneously by the two transmitters, in order to double the transmission rate. The strain sensor measured fractional particle displacement along the drill pipe’s *x* axis [4], whereas the triaxial accelerometer measured particle accelerations along *x*, *y*, and *z* axes [4] The strain sensor was used because of the smaller delay spread and therefore led to a less-frequency-selective behavior of strain channels [5], helping to improve data detection and reduce the bit error rate. The triaxial accelerometer was used to explore the performance of the three orthogonal acceleration channels for data detection. The frequency and impulse responses of all these channels were measured and studied, and are discussed in the next section. A schematic drawing of the entire testbed is provided in Figure 1c.

## 3. Experimental Results on Channel Measurements

Orthogonal frequency division multiplexing (OFDM) was used for signal transmission. Each actuator transmitted from 2 to 6 kHz, using 1024 sub-carriers, including 128 pilot tones for channel estimation and 96 null tones for noise power estimation. Each OFDM symbol duration was 256 ms, with a 25 ms guard time interval in between. A least squares method was used for channel estimation [7], whereas for data detection a minimum mean squared error (MMSE) algorithm was used (see the Appendix A). The used modulation and coding were quadrature phase shift keying (QPSK) and convolutional coding.

**Figure 2:**
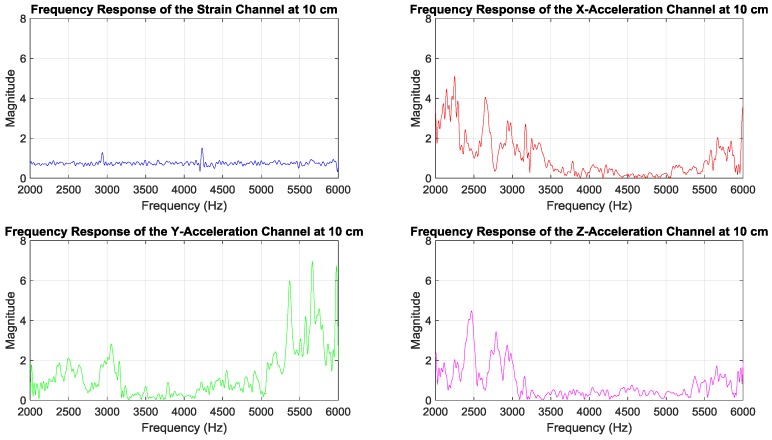
Magnitudes of frequency responses of channels measured by the receiving sensors in Figure 1b.

The magnitudes of channel frequency responses measured by the receiving sensors in Figure 1b are presented in Figure 2. It was observed that the strain channel had a nearly flat frequency response, whereas the acceleration channels’ frequency responses exhibited more frequency selectivity. The unequal strain and acceleration magnitudes in Figure 2 can be attributed to different sensor sensitivities: 50 mV/µε for the strain sensor and 10 mV/*g* for each channel of the triaxial accelerometer [4]. Here µε represents the strain magnitude unit in micro fractional particle displacement, whereas *g* = 9.8 m/s^2^ is the acceleration due to gravity, used as the unit for particle acceleration magnitude. A method is discussed in [4] to scale readouts of the strain and acceleration sensors according to their sensor sensitivities, such that strain and acceleration signal magnitudes can be compared using the same unit. In this paper, for simplicity, sensors readouts are used as they are for demodulating and detecting the transmitted data.

**Figure 3:**
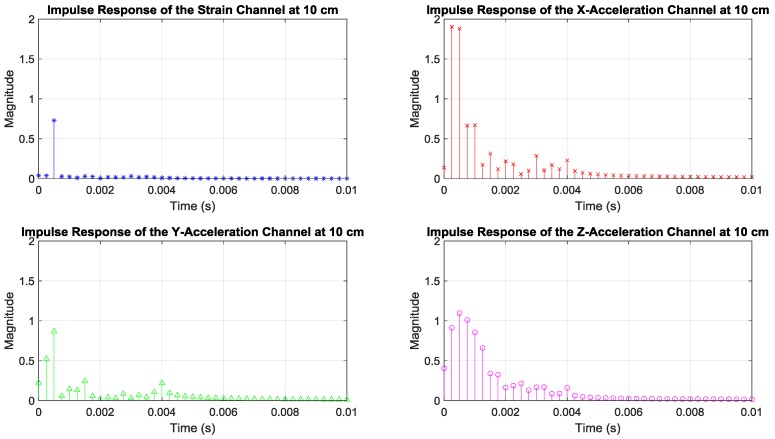
Magnitudes of impulse responses of the channels measured by the receiving sensors in Figure 1b.

To better understand the less-frequency-selective behavior of the strain channel compared to the acceleration channels, these channels were also examined in time domain by looking at their inverse Fourier transforms. The magnitudes of the channel impulse responses measured by the receiving sensors in Figure 1b are presented in Figure 3. Note that the strain channel impulse response had a shorter duration compared to the acceleration channels, behaviors that were also observed in References [4,5]. Given the properties of the Fourier transform, the short duration of the strain impulse response corroborates the relatively flat strain frequency response.

## 4. Experimental Results on Communication and Data Detection

In this section, communication results of one actuator transmitting one data stream are presented first. These will serve as benchmarks. Then, we present the communication results of two actuators transmit ting two data streams simultaneously in order to double the transmission rate over the same bandwidth.

### 4.1. One Actuator Transmitting One Data Stream

**Figure 4:**
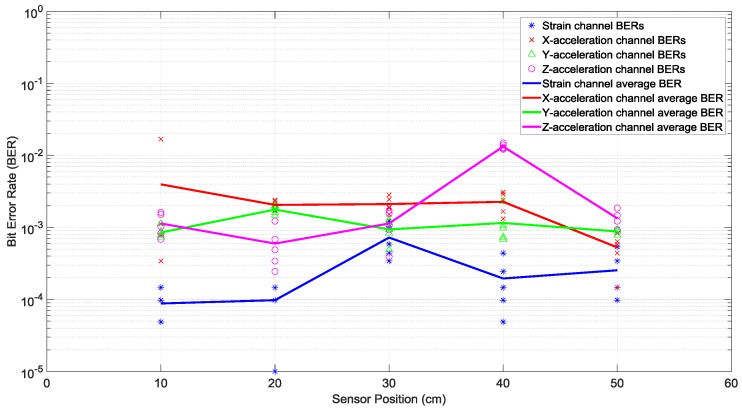
Bit error rates of various receiving sensors at different positions, with one actuator transmitting one data stream. The receivers were a strain sensor and a triaxial accelerometer with *x*, *y*, and *z* channels. The piecewise linear graphs represent the average BERs of each sensor versus the sensor position.

In this section, we consider the experiments where Tx 1 in Figure 1a transmitted fifty OFDM symbols in a row over the bandwidth of 2–6 kHz, repeated five times in order to have multiple trials. With the same transmit power per actuator throughout the entire paper and in all of the experiments, the measured bit error rates (BERs)—that is, bit error probabilities—for various receiving sensors at different positions identified in Figure 1b are presented in Figure 4. For each sensor at each position, five BERs and their average are provided, as obtained from five trials. It was observed that quite often the BERs of the strain sensor receiver were smaller than the BERs of the accelerometer receivers.

**Figure 5:**
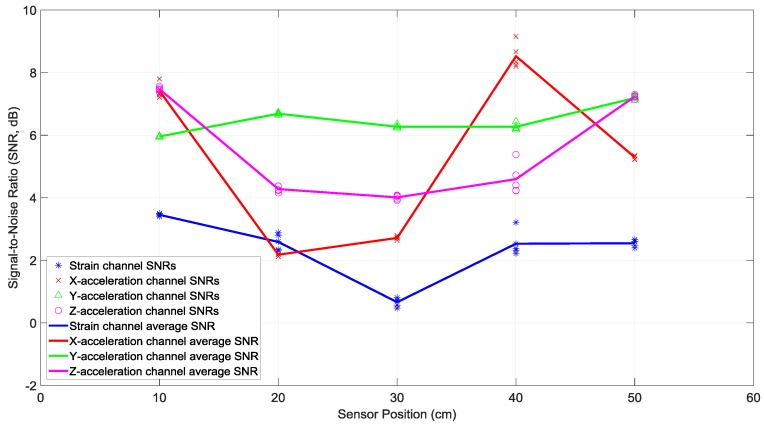
Signal-to-noise ratios of various receiving sensors at different positions, with one actuator transmitting one data stream. The receivers were a strain sensor and a triaxial accelerometer with *x*, *y*, and *z* channels. The piecewise linear graphs represent the average SNRs of each sensor versus the sensor position.

The signal-to-noise ratio (SNR) for each BER data point of Figure 4 and their average over five trials are provided in Figure 5. The SNRs reported throughout this paper were obtained by calculating the ratio of the power of pilot sub-carriers to the power of null sub-carriers [7]. In most cases, the SNRs of the strain sensor receiver were observed to be smaller than SNRs of the accelerometer receivers. This can be attributed to the sensitivity of the particular strain sensor used in the experiments, which produced weaker signals compared to the accelerometer signals, as discussed in the previous section.

The smaller BERs of the strain sensor receiver, while having smaller SNRs, can be related to the relatively flat strain channel frequency response in Figure 2. This makes equalization and data detection simpler and more accurate, compared to the non-flat and frequency-selective behavior of the acceleration channels’ frequency responses in Figure 2.

To better understand the data presented in Figure 4 and Figure 5, their measurement results averaged over five different receiver positions are listed in Table 1. It was observed that the BER of the strain sensor receiver was smaller than the BERs of the accelerometer receivers. This can be attributed to the relatively flat strain channel frequency response in Figure 2, which rendered equalization and data detection more accurate than the frequency-selective and non-flat acceleration channels’ frequency responses in Figure 2. The smaller SNR of the strain sensor receiver can be related to the sensitivity of the specific strain sensor used in the experiments, which produced weaker signals than the accelerometer signals, as mentioned in Section 3.

### 4.2. Two Actuators Simultaneously Transmitting Two Data Streams

**Figure 6:**
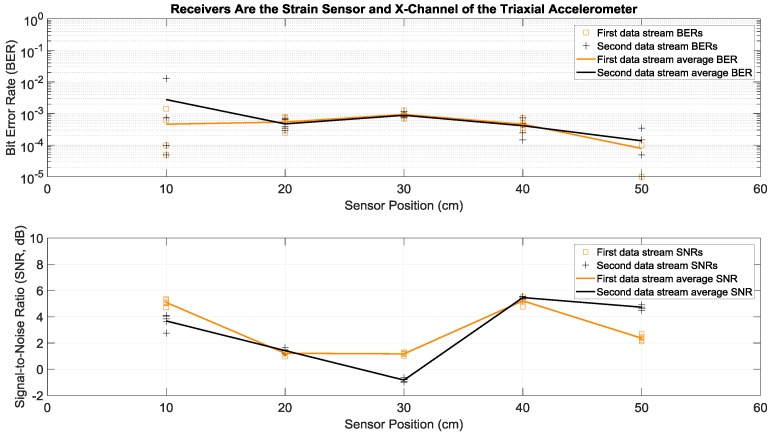
BERs (**top**) and SNRs (**bottom**) at different receiver positions, with two actuators transmitting two data streams simultaneously. The receivers were a strain sensor and the *x*-channel of a triaxial accelerometer. The piecewise linear graphs represent average BERs and SNRs for each of the two data streams versus the receiver position.

**Figure 7:**
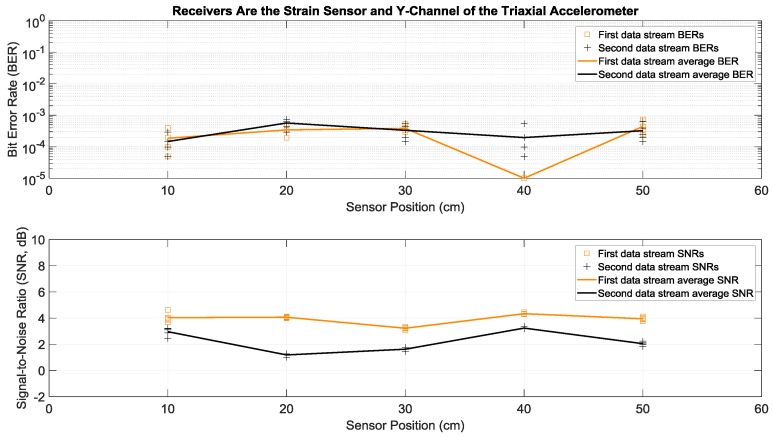
BERs (**top**) and SNRs (**bottom**) at different receiver positions, with two actuators transmitting two data streams simultaneously. The receivers were a strain sensor and the *y*-channel of a triaxial accelerometer. The piecewise linear graphs represent average BERs and SNRs for each of the two data streams versus the receiver position.

**Figure 8:**
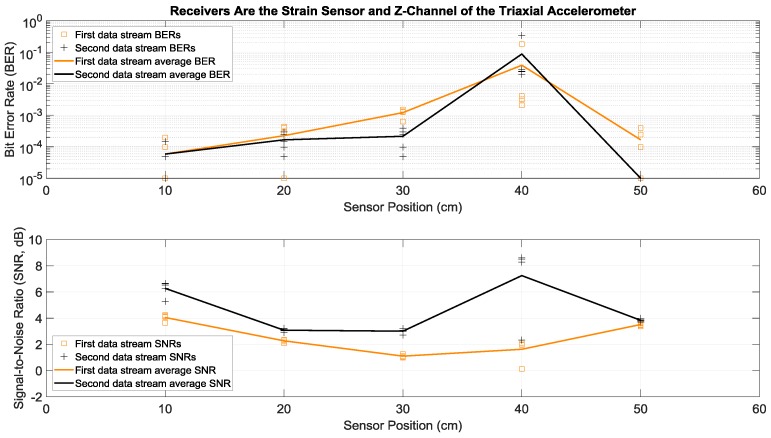
BERs (**top**) and SNRs (**bottom**) at different receiver positions, with two actuators transmitting two data streams simultaneously. The receivers were a strain sensor and the *z*-channel of a triaxial accelerometer. The piecewise linear graphs represent the average BERs and SNRs for each of the two data streams versus the receiver position.

**Figure 9:**
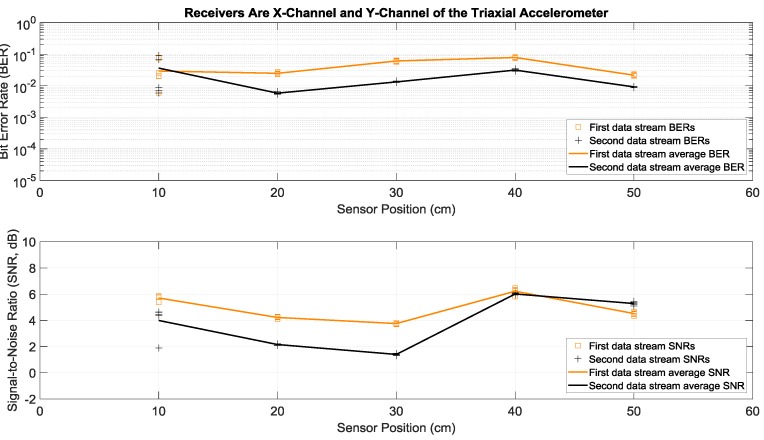
BERs (**top**) and SNRs (**bottom**) at different receiver positions, with two actuators transmitting two data streams simultaneously. The receivers were the *x*-channel and *y*-channel of a triaxial accelerometer. The piecewise linear graphs represent average BERs and SNRs for each of the two data streams versus the receiver position.

**Figure 10:**
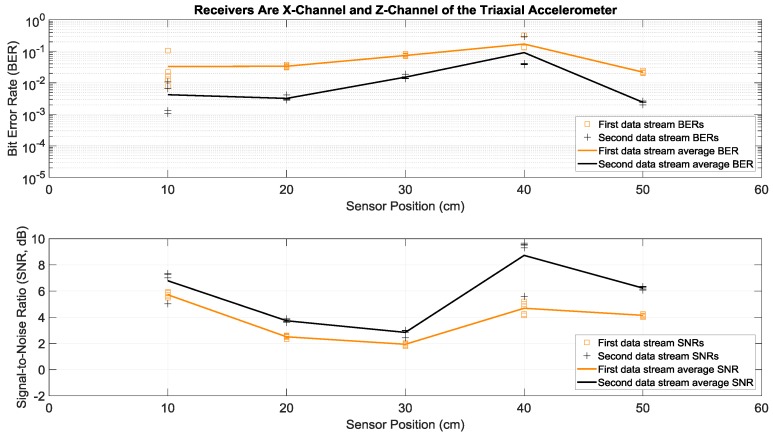
BERs (**top**) and SNRs (**bottom**) at different receiver positions, with two actuators transmitting two data streams simultaneously. The receivers were the *x*-channel and *z*-channel of a triaxial accelerometer. The piecewise linear graphs represent the average BERs and SNRs for each of the two data streams versus the receiver position.

**Figure 11:**
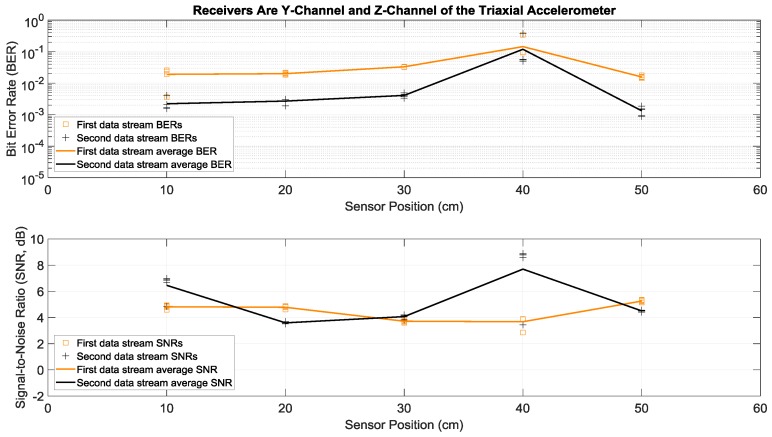
BERs (**top**) and SNRs (**bottom**) at different receiver positions, with two actuators transmitting two data streams simultaneously. The receivers were the *y*-channel and *z*-channel of a triaxial accelerometer. The piecewise linear graphs represent the average BERs and SNRs for each of the two data streams versus the receiver position.

Here we consider the experiments where Tx 1 and Tx 2 in Figure 1a both transmitted two different sets of data simultaneously and with the same power over the same bandwidth of 2 to 6 kHz. More specifically, each actuator transmitted fifty OFDM symbols in a row, repeated five times in order to have multiple trials. This simultaneous transmission of two data streams doubled the transmission rate, without any bandwidth increase. To separate and demodulate the two data streams at the receive side, two receiving sensors were used in this paper: the strain sensor and the triaxial accelerometer. Since the latter had the three *x*, *y*, and *z* acceleration channels, there were six possible receiver configurations using two channels: strain and *x*-acceleration, strain and *y*-acceleration, strain and *z*-acceleration, *x*-acceleration and *y*-acceleration, *x*-acceleration and *z*-acceleration, and *y*-acceleration and *z*-acceleration. Details of the data separation and detection method are discussed in the Appendix A. Measured BERs and SNRs for these six receivers at different positions identified in Figure 1b are presented in Figure 6, Figure 7, Figure 8, Figure 9, Figure 10 and Figure 11, respectively. For each receiver at each position, five BERs, five SNRs, and their averages for each of the first and the second data streams are provided, as obtained from five trials.

To compare performance of these six 2 × 2 systems with two transmitting actuators and a two-channel receiver, the best performance of the one actuator system of the previous subsection was considered as a benchmark. According to Figure 4, the lowest average BERs were below 10^−3^ for the system with the strain receiver, for average SNRs less than 4 dB at various receiver positions. Based on Figure 7 and compared to this benchmark, the two-actuator system using the strain and the *y*-acceleration receivers offered the best performance among the six two-actuator systems. This is because in all the receiver positions its average BERs were less than 10^−3^, with average SNRs close to or less than 4 dB. The second-best two-actuator system appeared to be the one which utilized the strain and the *x*-acceleration receivers, whose BERs and SNRs are shown in Figure 6.

Note that average BERs of the two-actuator systems that did not use the strain sensor receiver were all greater than 10^−3^ at all positions, for average SNRs ranging from 1.5 to 9 dB (see Figure 9, Figure 10 and Figure 11). As discussed in the previous section, this can be related to the relatively flat strain channel frequency response, which made equalization and data detection simpler and more accurate compared to the non-flat and frequency-selective behavior of the acceleration channels.

To better comprehend the data presented in Figure 6, Figure 7, Figure 8, Figure 9, Figure 10 and Figure 11, their measurement results averaged over five different receiver positions and over two data streams are provided in Table 2. It was observed that when the strain sensor was one of the receivers, the BER tended to be smaller. This held true even for strain and *z*-acceleration in Table 2, if the abnormally high BERs in Figure 8 for this receiver pair at 40 cm were not included in the average, which updated the BER and SNR for this receiver pair in Table 2 to 2.6 × 10^−4^ and 3.4 dB, respectively. Overall, these smaller BERs can be attributed to the relatively flat strain channel frequency response, which made data recovery and equalization simpler and more effective, compared to the acceleration channels which were non-flat and more frequency selective.

## 5. Conclusions

This paper demonstrates that by using two actuators, one can transmit two data sets simultaneously through drill strings and pipes in order to double the transmission rate in such communication media. The experimental bit error probability performance of the proposed two-actuator new system on a testbed was shown to be about the same as a system that used only one actuator, which can therefore offer only half of the data rate of the new system. Upon using more actuators, one can increase the data rate further.

Although only a two-channel receiver is proposed and used in this paper to separate and demodulate the data of the two actuators, one can use more receiving channels and sensors to improve the system performance. The benefit of having a multi-channel receiver with one actuator is discussed in References [4,6].

In this paper, various combinations of strain and acceleration sensors are considered at the receive side. Due to some properties of strain channels (e.g., smaller delay spreads [5] and their less-frequency-selective behavior) presented in this paper, it appears that a strain sensor receiver and an accelerometer receiver together can offer a good performance when separating and demodulating the two data streams transmitted simultaneously by the two actuators.

While not discussed in the paper, one can use the additional actuators to induce redundancy at the transmit side to reduce the bit error probability, using space-time codes and space-frequency codes.

## Figures and Tables

**Table 1:** Average BERs and SNRs of various receiving sensors, with one actuator transmitting one data stream.

Receiving Sensor	BER	SNR (dB)
Strain	2.7 × 10^−04^	2.4
X-Acceleration	2.2 × 10^−03^	5.2
Y-Acceleration	1.1 × 10^−03^	6.5
Z-Acceleration	3.5 × 10^−03^	5.5

**Table 2:** Average BERs and SNRs of various receiving sensor pairs, with two actuators simultaneously transmitting two data streams.

Receiving Sensor Pair	BER	SNR (dB)
Strain and *x*-acceleration	7.1 × 10^−04^	3.0
Strain and *y*-acceleration	2.9 × 10^−04^	3.1
Strain and *z*-acceleration	1.3 × 10^−02^	3.6
*x*- and *y*-acceleration	3.1 × 10^−02^	4.3
*x*- and *z*-acceleration	4.5 × 10^−02^	4.7
*y*- and *z*-acceleration	3.6 × 10^−02^	4.9

## Appendix A

Here we show how in the experiments the two data streams transmitted via OFDM by the two actuators were separated and detected using two received signals and an MMSE algorithm. The system model with $N_{rx}$ receivers and $N_{tx}$ transmitters can be written as

(A1) r=Hγ+n.

In the above equation for the κ-th OFDM data sub-carrier, $r={\left[r_{1}(f_{\kappa })\cdots r_{N_{rx}}(f_{\kappa })\right]}^{T}$ is the received signal vector, *^T^* is the transpose, $\gamma ={\left[\gamma_{1}(f_{\kappa })\cdots \gamma_{N_{tx}}(f_{\kappa })\right]}^{T}$ is the transmitted symbol vector, $n={\left[n_{1}(f_{\kappa })\cdots n_{N_{rx}}(f_{\kappa })\right]}^{T}$ is the noise vector, and H is the $N_{rx}\times N_{tx}$ channel matrix

(A2) $$H=\left[\begin{array}{ccc} H_{11}(f_{\kappa }) & \cdots & H_{1N_{tx}}(f_{\kappa })\\ \vdots & \ddots & \vdots \\ H_{N_{rx}1}(f_{\kappa }) & \cdots & H_{N_{rx}N_{tx}}(f_{\kappa }) \end{array}\right].$$

In the experiments, elements of γ were independent QPSK symbols, $N_{tx}=2$ corresponds to the two transmitting actuators, and $N_{rx}=2$ refers to any of these two receivers: strain and *x*-acceleration, strain and *y*-acceleration, strain and *z*-acceleration, *x*-acceleration and *y*-acceleration, *x*-acceleration and *z*-acceleration, and *y*-acceleration and *z*-acceleration. To recover the two transmitted symbols in γ at each sub-carrier from the corresponding 2×1 received signal vector r in (1), an MMSE detector [8] was used in the experiments

(A3) $$\hat{\gamma }={\hat{H}}^{H}{\left(\hat{H}{\hat{H}}^{H}+\hat{\Sigma }\right)}^{-1}r,$$

(A4) $$\hat{\Sigma }=\left[\begin{array}{ccc} {\hat{\sigma }}_{1}^{2} & \cdots & 0\\ \vdots & \ddots & \vdots \\ 0 & \cdots & {\hat{\sigma }}_{N_{rx}}^{2} \end{array}\right].$$

In Equation (3), $\hat{\gamma }$ includes the MMSE-based symbol estimates; $\hat{H}$ is the estimated channel matrix H in (2), obtained using a least squares method [7]; *^H^* is the transpose conjugate; and $\hat{\Sigma }$ in Equations (3) and (4) is the estimated $N_{rx}\times N_{rx}$ diagonal receiver noise covariance matrix, in which the noise variances are obtained using the null sub-carriers received by the receivers [7].

The above MMSE detector is a linear method, so it is highly desirable in practice due to its low computational complexity. Detection performance and BERs can be improved by using a nonlinear method such as a maximum likelihood method, but at the cost of higher computational complexity [8].
